# Modulation of Stem Cell Progeny by Probiotics during Regeneration of Gastric Mucosal Erosions

**DOI:** 10.3390/biology10070596

**Published:** 2021-06-28

**Authors:** Farah Al-Yassir, Ghalia Khoder, Subi Sugathan, Prashanth Saseedharan, Asma Al Menhali, Sherif M. Karam

**Affiliations:** 1 Department of Anatomy, College of Medicine and Health Sciences, United Arab Emirates University, Al Ain 17666, United Arab Emirates; fsa894@student.bau.edu.lb (F.A.-Y.); subisugathan@uaeu.ac.ae (S.S.); prash1987@hotmail.com (P.S.); 2 Department of Biological Sciences, Faculty of Science, Debbieh Campus, Beirut Arab University, P.O. Box 11-50-20 Riad El Solh 11072809, Beirut, Lebanon; 3 Department of Pharmaceutics and Pharmaceutical Technology, Sharjah Institute for Medical Research, College of Pharmacy, University of Sharjah, Sharjah 27272, United Arab Emirates; 4 Department of Biology, College of Science, United Arab Emirates University, Al Ain 15551, United Arab Emirates; 5 Zayed Research Center for Health Sciences, United Arab Emirates University, Al Ain 17666, United Arab Emirates

**Keywords:** probiotics, gastric erosion, cell proliferation, mucus, trefoil factor, H^+^,K^+^-ATPase

## Abstract

**Simple Summary:**

The wall of the stomach is easily damaged by different factors leading to serious diseases such as ulcers and cancer. In this study, the effects of a mixture of different types of beneficial bacteria (De Simone Formulation) were investigated in a mouse model of stomach wall damages induced by single or multiple doses of acetylsalicylic acid. Control mice received water. The stomachs of all mice were processed for microscopic examination and labeling of dividing stem cells and stomach cell lineages secreting mucus, acid, pepsin, and hormones. The results reveal beneficial effects for the multiple bacterial strain of De Simone Formulation given before or after the induction of stomach erosions. The regeneration of the stomach wall was associated with an increase in stem cell proliferation and enhanced production of protective factors such as mucus, and restoration of the aggressive factors produced by acid and pepsin secreting cell lineages. Therefore, the protective and therapeutic effects of the multi-strain healthy bacteria against stomach erosions involve modulation of not only dividing stem cells but also the multiple secretory cell lineages of the stomach.

**Abstract:**

Patients with gastric mucosal erosions are predisposed to chronic gastritis, ulcer or even cancer. The repair of mucosal erosions involves several events including proliferation of gastric epithelial stem cells. The aim of this study was to investigate the effects of the probiotic mixture of De Simone Formulation on gastric epithelial stem cell lineages in mouse models of gastric mucosal erosions. Gastric erosions were induced by a single oral gavage of 80% ethanol containing 15 mg/mL acetylsalicylic acid (5 mL/kg) following a daily dose of probiotic mixture (5 mg/day/mouse) for 10 days. In another protocol, erosions were induced by a daily gavage of acetylsalicylic acid (400 mg/kg/day/mouse) for 5 days before or after daily administration of probiotic mixture for 5 days. Control mice received water gavage for 10 days. All mice were injected with bromodeoxyuridine two hours before sacrifice to label S-phase cells. The stomachs of all mice were processed for histological examination, lectin binding, and immunohistochemical analysis. The results reveal that mice that received probiotics before or after the induction of erosion showed a decrease in erosion index with an increase in gastric epithelial stem/progenitor cell proliferation and enhanced production of mucus, trefoil factors, and ghrelin by mucous and enteroendocrine cell lineages. These mice also showed restoration of the amount of H^+^,K^+^-ATPase and pepsinogen involved in the production of the harsh acidic environment by parietal and chief cell lineages. In conclusion, this study demonstrates the beneficial effects of probiotics against gastric mucosal erosion and highlights the involvement and modulation of proliferative stem cells and their multiple glandular epithelial cell lineages.

## 1. Introduction

The single layer of glandular epithelial cells lining the stomach is highly vulnerable to several aggressive factors such as alcohol intake, non-steroidal anti-inflammatory drugs and *Helicobacter pylori* infection [1]. Therefore, gastric mucosal damages are very common and might evolve into serious issues ranging from chronic gastritis to gastric ulcer, and even cancer [2]. Antibiotics and proton pump inhibitors are commonly used to treat gastric mucosal damages. However, the emergence of antibiotic resistance [3] and the side effects of acid inhibitors [4,5] have drawn attention to the need for new therapeutic modalities. While several drugs and herbal extracts have shown promising results in the management of gastric mucosal damage [1,6], very little attention has been directed to the contribution of the regenerative capacity of gastric stem cells and few studies have assessed the effect of probiotics on gastric mucosal erosions.

Probiotics are microorganisms that exert beneficial effects on their hosts when ingested in sufficient amounts [7]. Probiotics are provided as mono-strain or multi-strain [8,9], such as the product originally called VSL#3 before 2016 or the recent De Simone Formulation (here referred to as DSF), which is a mixture of highly concentrated lyophilized living bacteria [10,11]. Probiotics have gained a great popularity in the prevention and treatment of several gastrointestinal diseases [12]. DSF has been extensively investigated and is currently recommended for the prevention and treatment of irritable bowel syndrome [13], pouchitis [14], Crohn’s disease [15], and ulcerative colitis [16]. Recently, DSF has also revealed a preventive effect in ulcerative colitis-associated carcinogenesis in mice [17].

We previously reviewed the extensive reports on the promising effects of probiotics on gastrointestinal disorders [18] and found only limited studies exploring the impact of probiotics on the gastric mucosa. Adverse physiological conditions of the stomach that attenuate the survival and growth of the probiotic microorganisms might have restricted this type of study. To overcome this limitation, treatment with multi-strain probiotics has been proposed [19]. In addition, studies have shown that multi-strain probiotics are more effective than mono-strain probiotics and have more successful chances of colonization [20].

For upper gastrointestinal disorders, it was reported that several individual probiotic bacteria including *Lactobacillus* spp. [21], *Lactobacillus rhamnosus* [22], *Lactobacillus gasseri* [23], *Lactobacillus acidophilus* [24], *Lactobacillus reuteri* [25] and *Bifidobacterium* [26] have promoted healing in different animal models of gastric ulcer. Similar findings were obtained when multi-strain probiotics were investigated [10]. Despite all the promising clinical and experimental findings, little is known about the cellular and molecular mechanisms of action of probiotics at the level of the gastric epithelial cell lineages. 

The normal gastric mucosa is lined by an epithelial cell layer organized to form numerous pits lined by mucous cells and is continuous with tubular glands populated with multiple cell lineages producing mucus, acid, pepsinogen and several hormones [27]. These cell lineages originate from proliferating epithelial stem cells. Recently, we reported that the administration of multi-strain probiotics DSF to healthy mice enhanced the production of trefoil factor (TFF) 1 and TFF2 by both types of gastric mucous cells and inhibited pepsinogen production by zymogenic cells [28]. In the current study, the aim was to investigate the glandular epithelial changes associated with the protective and therapeutic effects of DSF against gastric mucosal damage. Using a single dose of combined ethanol-acetylsalicylic acid (ASA or aspirin) administration, or repeated doses of ASA, gastric erosions were induced in mice and quantitative immunohistochemistry was employed to evaluate proliferating gastric epithelial stem/progenitor cells and their lineage descendants.

## 2. Materials and Methods

### 2.1. Animals

Thirty-nine, 3- to 5-month-old, male C57Bl/6 mice weighing 25 g on average were used in this study. All mice were given access to laboratory chow and water ad libitum and housed separately in clean, well ventilated cages under a 12 h/12 h light/dark cycle at room temperature (22–24 °C) for 30 days prior to experimentation. The animal protocol was designed to minimize pain and discomfort for the animals and was approved by the Animal Research Ethics Committee of the United Arab Emirates University (ERA_2016_5487).

### 2.2. Study Design 

**Figure 1:**
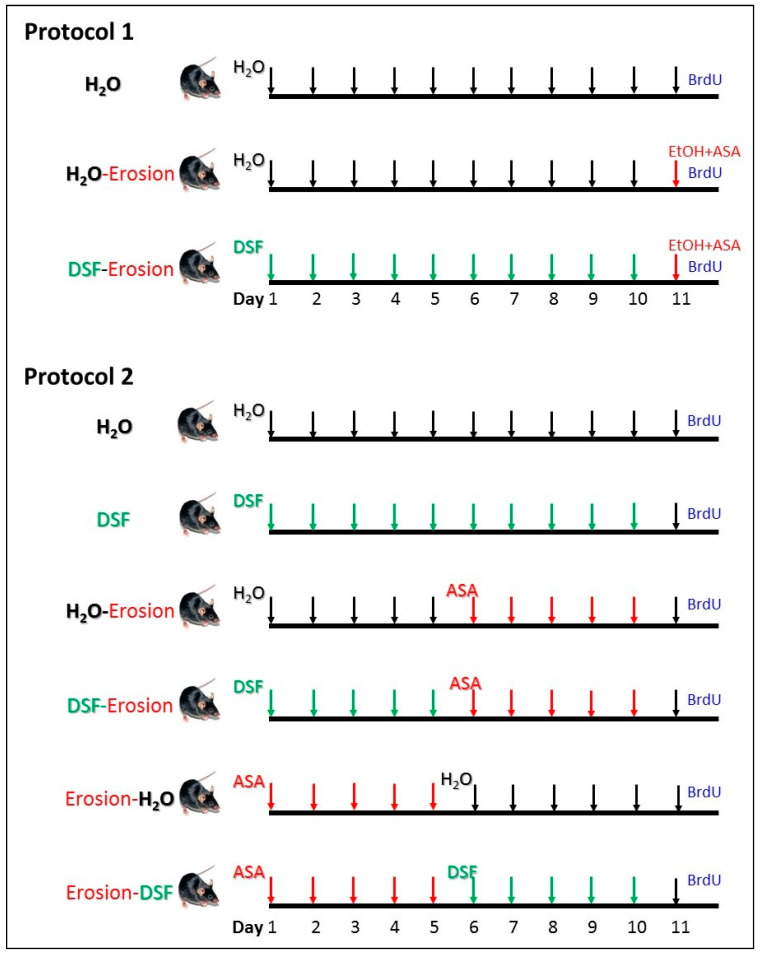
Design of the two experimental protocols carried out in this study. Protocol 1 was repeated in 3 experiments and each time, 3 littermate male mice were used (*n* = 9). They were labeled as H_2_O (control), H_2_O-erosion, and DSF-erosion. Protocol 2 was repeated in 5 experiments and each included 6 mice (*n* = 30) labeled as H_2_O, DSF, H_2_O-erosion, DSF-erosion, erosion-H_2_O, and erosion-DSF.

Two main protocols were independently carried out in this study (Figure 1). In the first protocol, 9 mice were used for three repeated experiments. Each experiment included 3 male weight-matched littermate mice. The first mouse served as a control and received a daily oral water gavage for 11 days. The second mouse received daily water gavage for 10 days and, to induce erosion, the last gavage of day 11 was 80% ethanol (5 mL/kg body weight) containing 15 mg/mL ASA 4-h before being euthanized [29]. The third mouse received a daily oral gavage of DSF (5 mg/day/mouse) for 10 days [28] and on day 11, gastric erosion was induced as in the second mouse using ASA-containing ethanol. 

The second protocol included 30 mice in 5 experiments. In each experiment, 6 matching littermate male mice from one or two mothers were used (Figure 1). Mice received 10 gavages of either water (H_2_O mouse), DSF at 5 mg/day (DSF mouse), water for 5 days followed by ASA at 400 mg/kg/day/mouse for 5 days (H_2_O-erosion mouse), DSF for 5 days followed by ASA for 5 days (DSF-erosion mouse), ASA for 5 days followed by water for 5 days (erosion-H_2_O mouse), and ASA for 5 days followed by DSF for an additional 5 days (erosion-DSF mouse). 

On day 11, dividing cells in the S-phase of the cell cycle were labeled in all mice of both protocols by a single intraperitoneal injection of 5′-bromo-2′-deoxyuridine (BrdU, 120 mg/kg body weight), and two hours later, the mice were euthanized under anesthesia and the stomachs were immediately collected and processed together from each experiment of each protocol for histological, lectin binding, and immunohistochemical analysis. The body weights of all mice of both protocols were measured on days 1, 6 and 11 using a digital scale and the food intake during day 10 was also estimated [28]. 

The DSF used in this study was the same original formula of VSL#3 which we previously used [28] and was composed of a mixture of highly concentrated lyophilized living bacteria (450 billion bacteria per sachet) of four species of Lactobacilli: *Lactobacillus acidophilus* (DSM 24735), *Lactobacillus paracasei* (DSM 24734), *Lactobacillus plantarum* (DSM 24730), and *Lactobacillus delbrueckii Subsp. Bulgaricus* (DSM 24734); three species of Bifidobacteria: *Bifidobacterium infantis* (DSM 24737), *Bifidobacterium longum* (DSM 24736), and *Bifidobacterium breve* (DSM 24732) and one species of *Streptococcus salivarius subsp. thermophilus* (DSM 24731).

### 2.3. Macroscopic Assessment of Gastric Erosions

The stomachs of all mice were immediately cut-open along the greater curvature, and gently washed with ice-cold phosphate-buffered saline (PBS) to clean off any food residue or blood clots. For each stomach, the total surface area of the glandular region was measured using ImageJ software v1.53j originally developed by Wayne Rasband, (National Institutes of Health, Bethesda, MD, USA). Then, the total surface area of visible erosions was also measured to calculate the gastric erosion index.

### 2.4. Microscopic Assessments of Gastric Mucosal Tissues

Tissue samples were processed for histological, lectin histochemical and immunohistochemical analyses according to our previously published methods [28]. The stomachs of all mice were immediately immersed overnight in Bouin’s solution and then processed for paraffin embedding. To ensure equal conditions and minimize variations, stomach tissues of each 3 littermate mice (control, erosion, and DSF-erosion) of the first protocol or 6 littermate mice (H_2_O, DSF, H_2_O-erosion, DSF-erosion, erosion-H_2_O, and erosion-DSF) of the second protocol were embedded together in the same paraffin block. Longitudinal tissue sections (5-µm-thick) near the greater curvature were stained with hematoxylin and periodic acid Schiff (PAS) solutions (Abcam, Cambridge, UK) and examined with an Olympus microscope connected to DP70 digital camera. The gastric mucosal thickness was measured in comparable areas of the corpus using the scale bar tool of the DP70 camera software. For each stomach, three measurements from areas separated by 10 glands were obtained and averaged.

To label surface and neck mucous cells, tissue sections were deparaffinized, rehydrated, and incubated with a blocking solution containing 1% bovine serum albumin and 0.5% tween-20 in PBS for 45 min at room temperature, and then incubated in fucose-specific *Ulex europaeus* agglutinin I (UEA) lectin (Vector Laboratories, Burlingame, CA, USA) conjugated to rhodamine for 1 hr. Following PBS washes, tissues were incubated for 1 hr with *N*-acetyl-d-glucosamine-specific *Griffonia simplicifolia* II (GS) lectin (Thermo-Fisher Scientific, Molecular probes by Life Technologies, Oregon, OR, USA) conjugated to fluorescein isothiocyanate. The tissue sections were washed in PBS and mounted with fluoro-shield mounting medium with 4′,6-diamidino-2-phenylindole (Abcam, Cambridge, UK).

For immunohistochemistry, tissue sections were deparaffinized, rehydrated and washed in PBS. The endogenous peroxidase activity was blocked by incubating tissue sections in 3% hydrogen peroxide for 35 min. All tissue sections on the slide were circled with a hydrophobic film using a PAP pen (Dako, Glostrup, Denmark). To Block non-specific binding sites, the sections were incubated in PBS containing 1% bovine serum albumin and 0.5% Tween-20 for 45 min. The tissue sections were then incubated for 1 h using mouse monoclonal anti-BrdU antibody (Medical and Biological Laboratories Co., Nagoya, Japan) as a marker for proliferating cells or for overnight at 4 °C with mouse monoclonal anti-pepsinogen C (Abcam, Cambridge, UK) as marker for chief cells, or anti-H^+^,K^+^-ATPase β-subunit (Medical and Biological Laboratories Co. Woburn, MA, USA) for parietal cells, or anti-ghrelin antibodies for enteroendocrine cells, a kind gift from Catherine Tomasetto, raised in the IGBMC laboratories, Strasbourg, France [30], or rabbit polyclonal anti-TFF1 and anti-TFF2 antibodies (IGBMC, France), respectively, for mucous pit and neck cells [31]. Following PBS washes, the tissue sections were incubated with biotinylated donkey anti-mouse or anti-rabbit immunoglobulin G for 1 hr. Tissues were washed in PBS and then incubated in peroxidase-conjugated extravidin (Sigma, St. Louis, MO, USA). The antigen-antibody binding sites were revealed by using 3,3′-diaminobenzidine tetrahydrochloride (Sigma, St. Louis, MO, USA). Since these tissues were processed and embedded together in the same paraffin block and then sectioned together and mounted on the same slide, the beginning of color development in one section compared to others might indicate a higher level of proteins. For example, this was the case for the slide probed with anti-H^+^,K^+^-ATPase where the brown color started to appear first in the erosion-induced tissues and few seconds later in the two other tissues, control and DSF-erosion.

### 2.5. Quantification

To estimate the number of cells immunolabeled with antibodies specific to BrdU or ghrelin, the Fiji ImageJ software was used. At least three JPEG images obtained at 20× objective with the best longitudinal orientation of gastric glands were examined in each tissue section of the three or six groups of mice. In each image, the numbers of cells per 10 glands or per field were expressed as the mean ± SE. 

To estimate the labeling intensities of UEA or TFF1 in pit cells, GS or TFF2 in neck cells, pepsinogen in zymogenic cells and H^+^,K^+^-ATPase in parietal cells, images obtained from tissue sections probed with lectins or antibodies were loaded into the ImageJ software. In the case of immunoperoxidase labeling, image deconvolution was used to separate the DAB staining from the hematoxylin and/or PAS staining. Images were then converted to 8-bit and pixel density was calculated using the analysis tool. The quantification was carried out considering only the areas of intact mucosa as many cells were absent in the areas of erosions. The percentage values of labeled pixels obtained from the software were taken to reflect the amount of mucus/peptide/protein in the cells analyzed. Data were presented as mean or fold change relative to control ± SE.

### 2.6. Statistical Analyses

All data were presented as mean ± SE. The significance of differences between body weight gain, lectin binding and immunolabeling of the 3 groups of first protocol (*n* = 3 per group) and the 6 groups of the second protocol (*n* = 5 per group) were analyzed by one way analysis of variance (ANOVA) followed by Tukey’s post hoc test to determine differences between particular pairs of experimental and control groups. The difference between the gastric erosion indices of H_2_O-erosion (*n* = 3) and DSF-erosion (*n* = 3) of the first protocol was determined by using the Student’s t-test. All results were analyzed using GraphPad Prism v9.1.2.226 (GraphPad Software, Inc., San Diego, CA, USA). *p* < 0.05 were considered statistically significant.

## 3. Results

### 3.1. Effects of DSF on Gastric Erosions Induced by a Single Gavage of ASA-Containing Ethanol

**Figure 2:**
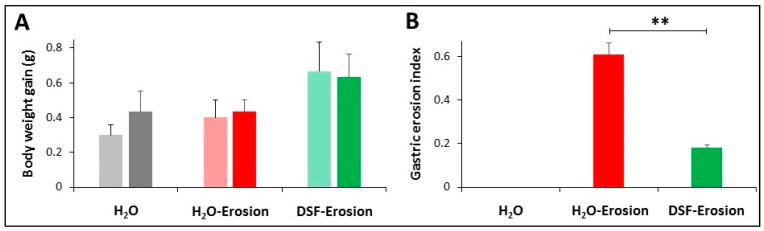
Body weight gain (**A**) and gastric erosion index (**B**) in control (H_2_O), erosion-induced, and DSF-pretreated then, erosion-induced mice. The body weight gain was measured after 6 and 11 days of starting the experiment. Data from 3 mice in each group are presented as mean ± SE. Differences in body weight gain among the three groups of mice were not statistically significant. Gastric erosion index is reduced in mice given DSF for 10 days and then ASA-containing ethanol on day 11. This reduction was statistically significant when compared with the erosion-induced group (** *p* <0.01). Data are presented as mean ± SE.

**Figure 3:**
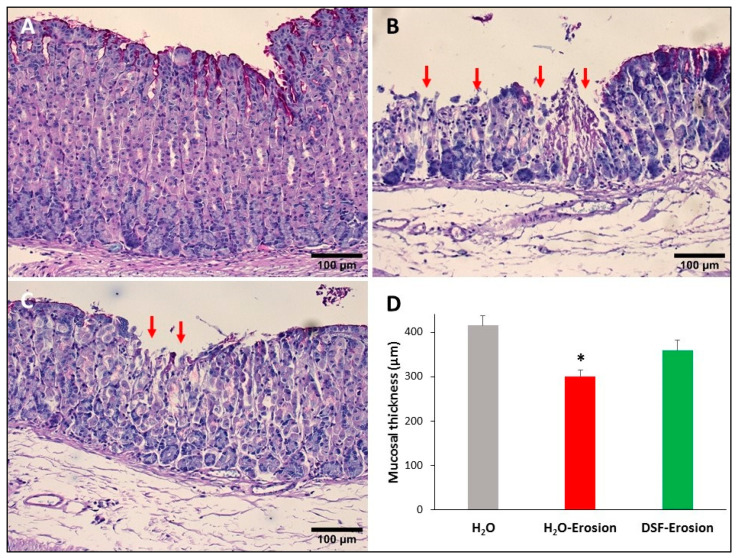
PAS-stained gastric mucosae of H_2_O control (**A**), erosion-induced (**B**) and DSF-pretreated then erosion-induced (**C**) mice. The red arrows in (**B**,**C**) are pointing to mucosal erosions. Note that the area of erosion is smaller in (**C**) than in (**B**). (**D**) Measurements of the gastric mucosal thickness in control, erosion-induced and DSF-pretreated erosion-induced mice. Data are presented from 3 animals per group as mean ± SE. Comparing the groups shows significance only between H_2_O-erosion and the H_2_O control. * *p* < 0.05; scale bar = 100 µm.

**Figure 4:**
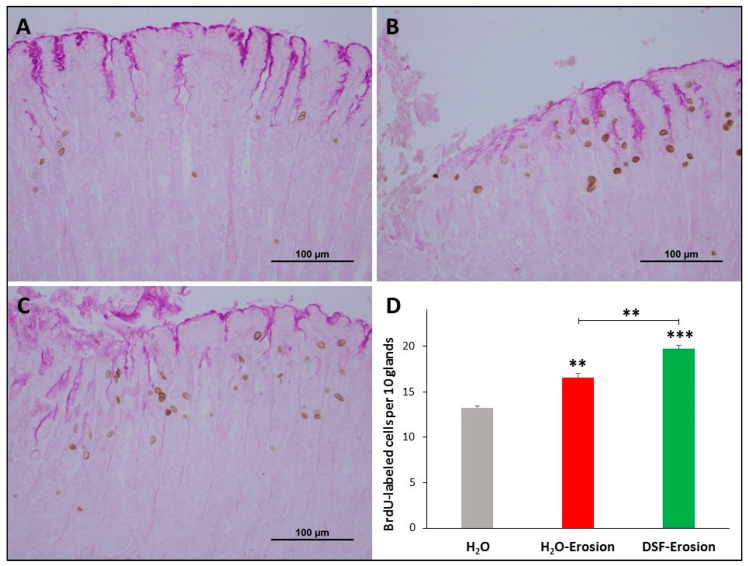
BrdU labeling in the gastric mucosae of H_2_O control (**A**), erosion-induced (**B**) and DSF-pretreated then erosion-induced (**C**) mice. The tissues were counter-stained with PAS to show mucus-secreting pit cells at the luminal surface. (**D**) Counts of the BrdU-labeled cells in control, erosion-induced and DSF-pretreated then erosion-induced mice (*n* = 3 per group) are presented as mean ± SE. In this figure and all following figures, asterisks above columns indicate significant difference when the value of that column is compared with the H_2_O control. Asterisks above the line indicate differences between adjacent groups ** *p* < 0.01, *** *p* < 0.001; scale bar = 100 µm.

**Figure 5:**
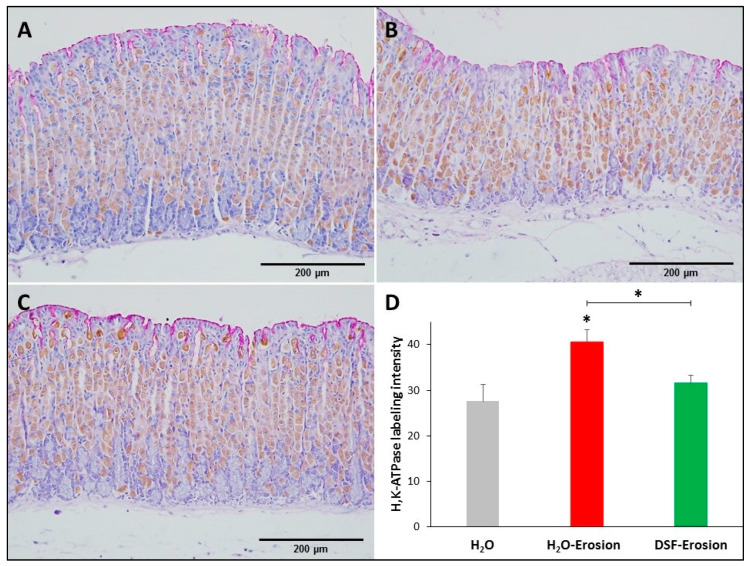
H^+^,K^+^-ATPase immunolabeling in the gastric mucosae of H_2_O control (**A**), erosion-induced (**B**) and DSF-pretreated then erosion-induced (**C**) mice. The tissue sections were counterstained with hematoxylin and PAS. Specific antibodies against H^+^,K^+^-ATPase β-subunit were used to label parietal cells distributed throughout the gastric glands. (**D**) Quantification of H^+^,K^+^-ATPase labeling intensity per field is presented from three mice in each group as mean ± SE. * *p* < 0.05; scale bar = 200 µm.

**Figure 6:**
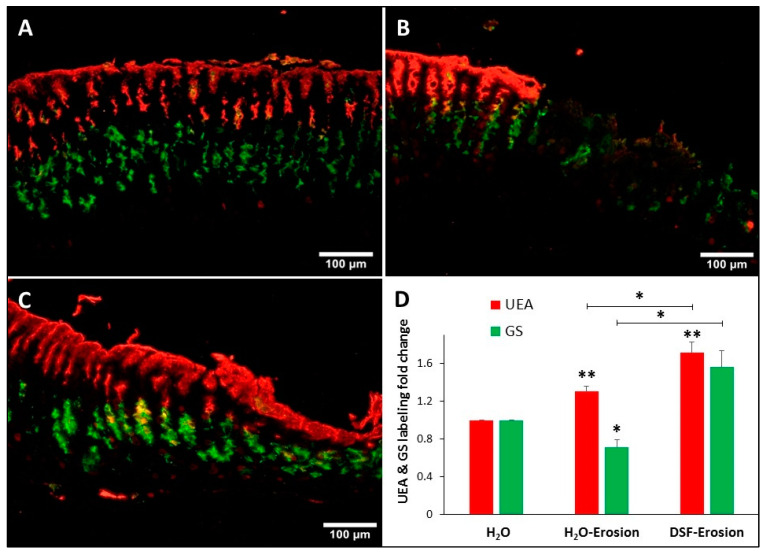
Double lectin histochemistry of the gastric mucosa of H_2_O control (**A**), erosion-induced (**B**) and DSF-erosion (**C**) mice. Stomach tissue sections were incubated with rhodamine-conjugated UEA-I lectin (red) and GSII-FITC (Green). UEA-I demarcates the surface mucous cells lining the luminal surface and the mucosal pits. GSII lectin defines the mucous neck cells in the middle of the mucosa. (**D**) Fold change in the levels of UEA-I and GSII labeling intensity of control (*n* = 3), gastric lesion (*n* = 3) and DSF-erosion (*n* = 3) tissues (**D**) are presented as mean ± SE. * *p* < 0.05, ** *p* < 0.01; scale bar = 100 µm.

**Figure 7:**
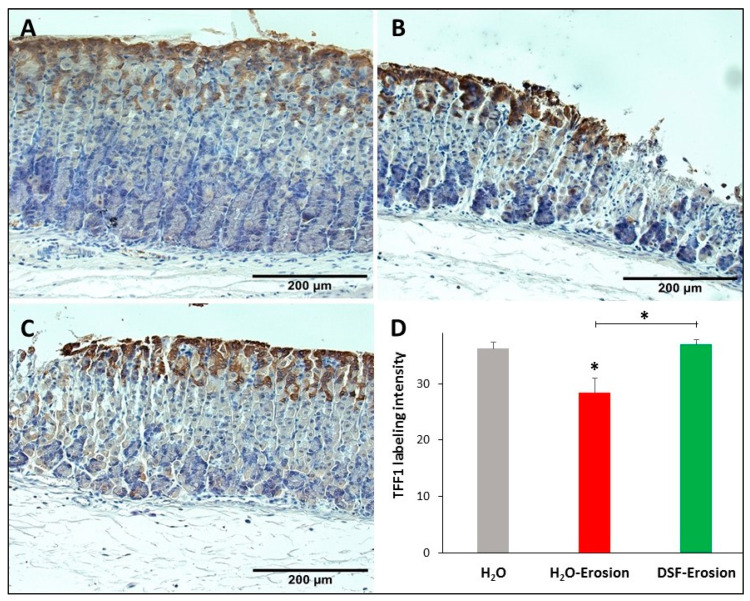
TFF1 immunolabeling in the gastric mucosae of H_2_O control (**A**), erosion-induced (**B**) and DSF-erosion (**C**) mice. The tissue sections were counterstained with hematoxylin. Brownish TFF1-labeled cells are seen at the luminal surface and in the pit regions. (**D**) Quantifications of TFF1 labeling intensity is calculated from three mice in each group and presented as mean ± SE. * *p* < 0.05; scale bar = 200 µm.

**Figure 8:**
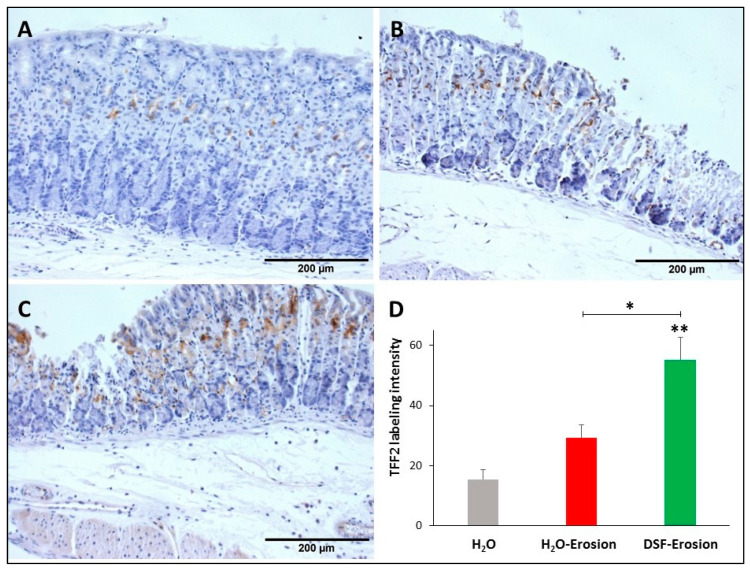
TFF2 immunolabeling in the gastric mucosae of H_2_O control (**A**), erosion-induced (**B**) and DSF-pretreated (**C**) mice. The tissue sections were counterstained with hematoxylin. Brownish TFF2-labeled cells are seen in the middle of the gastric mucosae. Labeled cells are more apparent in DSF-pretreated tissues than in control and erosion-induced tissues. (**D**) Quantification of TFF2 labeling intensity per field is presented from three mice in each group as mean ± SE. * *p* < 0.05 and ** *p* < 0.01; scale bar = 200 µm.

**Figure 9:**
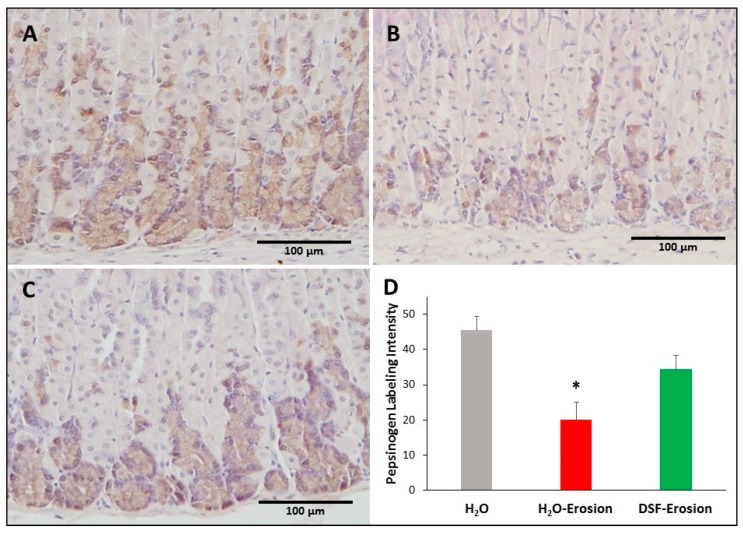
Pepsinogen immunolabeling in the gastric mucosae of H_2_O control (**A**), erosion-induced (**B**) and DSF-erosion (**C**) mice. The tissue sections were counterstained with hematoxylin. Brownish pepsinogen-labeled cells are seen in the basal regions of the gastric mucosae. Labeled cells are more prominent in control and DSF-pretreated tissues than in erosion-induced tissues. (**D**) Quantification of pepsinogen labeling intensity (or percentage of labeled pixels in equivalent areas) of the mucosa of three mice in each group is presented as mean ± SE. * *p* < 0.05; scale bar = 100 µm.

**Figure 10:**
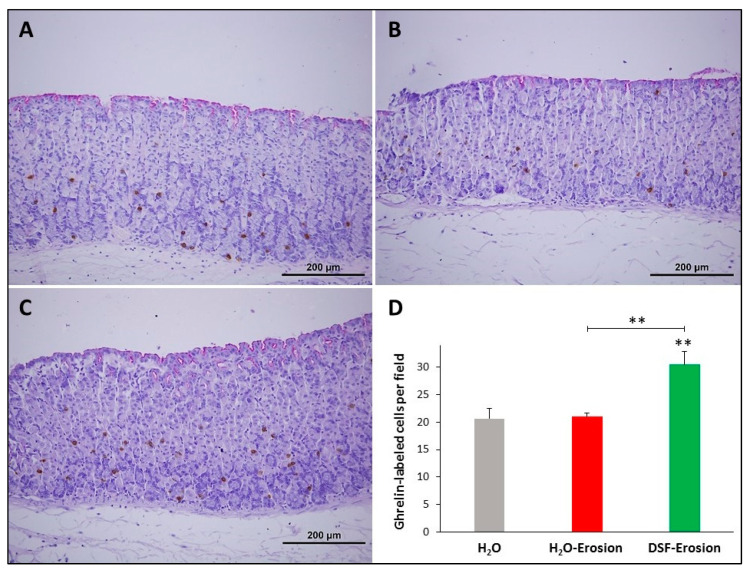
Ghrelin immunolabeling in the gastric mucosae of H_2_O control (**A**), erosion-induced (**B**) and DSF-erosion (**C**) mice. The tissue sections were counterstained with PAS and hematoxylin. Small brownish ghrelin-labeled cells are seen scattered mostly in the basal regions of the gastric mucosae. (**D**) Quantification of ghrelin-labeled cells per field of the mucosa of three mice in each group is presented as mean ± SE. ** *p* < 0.01; scale bar = 200 µm.

The body weight and food consumption of all mice on experimental days 6 and 11 did not reveal any significant difference between the groups (Figure 2A). However, analysis of gastric mucosal tissues revealed several significant macroscopic, microscopic, and molecular observations due to changes in different epithelial cell lineages of DSF-pretreated mice when compared to mice of the two other groups as described below (Figure 2B, Figure 3, Figure 4, Figure 5, Figure 6, Figure 7, Figure 8, Figure 9 and Figure 10).

#### 3.1.1. Probiotics Attenuate Induction of Gastric Mucosal Erosions

Examination of the luminal surfaces of the stomachs of the three groups of mice revealed scattered small erosive lesions located mainly in the ethanol/ASA group. Only a few smaller erosions were observed in the stomachs of the DSF group. While the erosion index in the erosion-induced group was 0.61 ± 0.06, it was only 0.18 ± 0.01 in the DSF-pretreated group. This difference was statistically significant (*p* < 0.01; Figure 2B). Therefore, the percentage of the inhibition of gastric erosion was estimated at 69.1%.

Microscopic examination of stomach tissue sections stained with conventional PAS and hematoxylin showed apparent structural differences when control tissues were compared to the erosion-induced and DSF-pretreated tissues. The corpus regions of control stomachs had intact surface epithelium connected to tubular glands with large scattered parietal cells, basal basophilic zymogenic cells, and PAS-positive mucous cells at the luminal surface and pits (Figure 3A). In contrast, the stomachs of the ethanol/ASA-treated mice exhibited scattered areas of mucosal damages. In addition to the cellular necrosis, the glandular cells were disorganized (Figure 3B). Interestingly, DSF-pretreated tissues showed fewer and smaller areas of necrosis (when compared to erosion-induced mice) and glandular integrity was mostly maintained (Figure 3C). Moreover, measurements of the glandular mucosal thickness in a comparable region of tissue sections showed values that varied from 372 to 428 µm in control, 277 to 322 µm gastric erosion group and 317 to 398 µm in DSF-pretreated tissues (Figure 3D). The glandular mucosal thickness in the DSF-pretreated tissues was comparable to the control tissues, but a statistically significant difference was found between the erosion and control groups (*p* < 0.05, Figure 3D). In addition, systematic examination of several molecular cell markers using a variety of microscopy approaches demonstrated several differences in the epithelial cell lineages.

#### 3.1.2. DSF-Pretreatment Stimulates Gastric Stem/Progenitor Cell Proliferation

To correlate the morphological changes with cell proliferation, BrdU-labeled S-phase cells were evaluated in the three groups of mice. The gastric mucosae of control mice showed a few BrdU-labeled cells at the pit-gland junction close to the luminal surface (Figure 4A). However, the area of the pit-gland junction tended to show more BrdU-labeled cells in the intact areas of erosion-induced and DSF-pretreated tissues (Figure 4B,C). Moreover, the glandular region in which BrdU-labeled cells are located in DSF-pretreated tissues appeared wider than that of the erosion-induced group and even more expanded when compared with control tissues (Figure 4A–C). Quantification of BrdU-labeled cells in three different micrographs obtained from equivalent oxyntic mucosal regions of control, erosion-induced, and DSF-pretreated tissues revealed the presence of 13.3 ± 0.2, 16.6 ± 0.4 and 19.7 ± 0.4 cells per 10 glands, respectively (Figure 4D). Data analysis showed significant increase in proliferating cells in DSF-erosion tissues when compared with the erosion-induced (*p* < 0.01) or control (*p* < 0.001) tissues (Figure 4D).

#### 3.1.3. DSF-Pretreatment Restores Normal Level of H^+^,K^+^-ATPase in Parietal Cells

Microscopic examination showed that the H^+^,K^+^-ATPase labeling appeared more intense in the erosion-induced tissues than in the control and DSF-treated tissues (Figure 5A–C). Quantification of the percentage of pixels in labeled areas of images captured with 20× objective revealed that the increased H^+^,K^+^-ATPase labeling was significant in erosion-induced tissue when compared with control and DSF-erosion tissues (* *p* < 0.05; Figure 5D). 

#### 3.1.4. DSF-Pretreatment Increases Lectin- and Immuno-Labeling of Mucous Cells

Double lectin histochemistry of the gastric mucosae of control, erosion-induced and DSF-erosion mice was used to label and evaluate the amounts of glycosylated mucins in both pit and neck mucous cells (Figure 6A–C). Analysis of lectin labeling intensities in erosion-induced tissues as compared to control tissues revealed a significant increase in UEA labeling (*p* = 0.043), but a decreased GS labeling (Figure 6D). When the DSF-pretreated tissues were compared to control tissues and erosion-induced tissues, there was an increasing trend in both UEA and GS labeling (Figure 6C). Measurements of UEA labeling intensities revealed a significant increase in DSF-pretreated tissues when compared to either control or erosion-induced tissues (*p* = 0.005 and 0.048, respectively). Pretreatment with DSF also induced an increase in the levels of intensity of GS-labeling of neck cells when compared to gastric erosion tissues (*p* = 0.021), but not to control tissues (*p* = 0.056). 

Because both types of mucous cells synthesize and secrete TFFs, gastric tissues of the three groups of mice were probed with antibodies specific to TFF1 and TFF2. TFF1 was localized in the pit cells lining the luminal surface and along the pit regions (Figure 7A–C). Quantification of the TFF1 labeling intensity (or the percentage of pixels of labeled areas) in images obtained with 20× magnification revealed a decrease in TFF1-labeling in the erosion-induced tissues as compared to control (*p* = 0.047). Comparing TFF1-labeling intensities in DSF-pretreated tissues to erosion-induced, but not control tissues, showed a significant increase, *p* = 0.035 (Figure 7D). 

Similarly, stomach tissue sections from the three groups of mice were probed with anti-TFF2 antibodies in order to determine any implication of DSF pretreatment on TFF2 production by mucous neck cells. Immunolabeling demonstrated the presence of TFF2 in the neck region of the three tissues with a tendency to be more prominent in DSF-pretreated tissues (Figure 8A–C). Quantification of the percentages of pixels in the labeled areas of images obtained with 20× revealed a significant increase in the intensity of TFF2 labeling of DSF-pretreated tissues when compared to either erosion-induced (*p* = 0.037) or control tissues (*p* = 0.008; Figure 8D). However, the difference was not significant when erosion-induced tissues were compared with control (*p* = 0.063; Figure 8D).

#### 3.1.5. DSF Pretreatment Reduces Pepsinogen Production by Zymogenic Cells 

To test whether increased GS-labeling and TFF2 in mucous neck cells of DSF-pretreated tissues was associated with changes in their progeny, the zymogenic cells, tissues from all mice were probed with antibodies specific to pepsinogen. There was some variability in the immunolabeling pattern of pepsinogen with a tendency to find less labeling in the erosion-induced tissues (Figure 9A–C). Quantification of the percentage of pixels in labeled areas of images obtained with 20× magnification revealed a significant decrease in the pepsinogen labeling intensity in the erosion-induced tissues when compared to control (*p* = 0.029). The pepsinogen intensity in DSF-pretreated tissues was not significantly different, neither from erosion-induced nor control tissues (*p* = 0.139 and 0.177, respectively; Figure 9D).

#### 3.1.6. DSF Pretreatment Increases Ghrelin-Secreting Enteroendocrine Cells 

To evaluate the impact of DSF pretreatment on ghrelin-secreting enteroendocrine cells, ghrelin antibodies were used since they label different subtypes of endocrine cells in the gastric mucosa [30]. Immunoprobed tissue sections of control, erosion-induced and DSF-erosion mice revealed a similar scattered pattern of distribution of ghrelin-secreting cells predominating in the basal region of gastric glands (Figure 10A–C). However, tissue sections obtained from the DSF-erosion mice showed more immuno-labeled cell counts per field than in the control and erosion tissues (Figure 10D). On average, there were 21 ± 1.8, 21 ± 0.7 and 31.03 ± 2.4 cells per field of a gastric mucosal tissue section of control, erosion-induced and DSF-erosion mice, respectively (Figure 10D). This difference was statistically significant only in DSF-erosion group when compared to control (*p* = 0.009) and erosion-induced groups (*p* = 0.007).

Taken together, these data demonstrated that DSF pretreatment plays an essential protective role in ethanol/ASA-induced gastric erosion. The injured mucosa of DSF-pretreated mice acquired significant inhibition of gastric erosion and maintained most of the gastric mucosal integrity due to (i) increased gastric epithelial cell proliferation, (ii) upregulation of mucus and associated TFFs, (iii) restoration in cellular H^+^,K^+^-ATPase and pepsinogen, and (iv) increased number of ghrelin-secreting cells.

### 3.2. Effects of DSF on Gastric Erosion Induced by Multiple ASA Gavages

**Figure 11:**
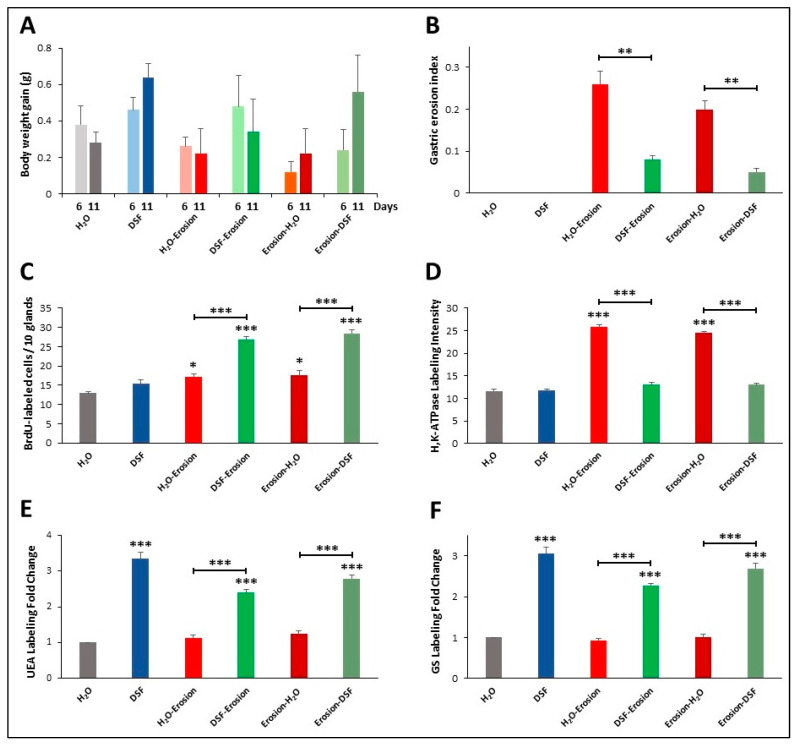
Characterization of the 6 groups of mice of the second protocol of multiple-ASA-gavage erosion model. The graphs show body weight gain (**A**), gastric erosion index (**B**), BrdU-labeling (**C**), H^+^,K^+^-ATPase immunolabeling intensity (**D**), UEA lectin labeling (**E**) and GS lectin labeling (**F**). In all graphs, data are presented for each group of 5 mice as mean ± SE. Asterisks above the columns compare the value of that column with the H_2_O control group and on the horizontal lines compare adjacent paired groups. * *p* < 0.05, ** *p* < 0.01, *** *p* < 0.001.

In the second protocol of daily ASA gavage for 5 successive days, there were no significant differences in body weight gain of the groups of mice on days 6 and 11 (Figure 11A). However, the erosion index in the erosion-induced mice was significantly reduced (*p* < 0.001) from 0.26 ± 0.03 to 0.08 ± 0.01 in DSF-erosion group and from 0.20 ± 0.02 in erosion-H_2_O mice to 0.05 ± 0.01 in erosion-DSF group (*p* < 0.01) (Figure 11B). The percentages of inhibition of erosion indices were about 70% for erosion-DSF and 67% for DSF-erosion groups. As for the S-phase BrdU-labeled cells, their numbers were significantly increased (*p* < 0.001) in erosion-DSF, and DSF-erosion groups compared to their control groups (Figure 11C). In addition, quantification of the percentage of pixels in H^+^,K^+^-ATPase-labeled areas of images captured with 20× objective revealed significantly increased labeling in erosion-induced tissue (Figure 11D) when compared with H_2_O control, erosion-DSF, and DSF-erosion tissues (*p* < 0.001). 

To assess the effects of DSF gavages on mucous cell lineages of erosion induced mice, double lectin histochemistry was used. There was an increasing trend in both UEA and GS labeling of DSF-pre- or post-erosion induced tissues. Measurements of fold changes of lectin labeling intensities revealed that the increase in both UEA and GS labeling in DSF-pre- and post-treated tissues were statistically significant compared to H_2_O control tissues and erosion-induced tissues (Figure 11E,F).

## 4. Discussion

There is an increasing interest in pre-clinical and clinical studies on the use of probiotics for gastrointestinal disorders. Some reports have demonstrated the beneficial effects of the probiotics on modulating the gut microbiota [32,33], cell proliferation [34,35], and mucus secretion [36,37]. However, the cellular effects involved in the gastric mucosal protection are not well explored. Our previous study has shown several effects for the same probiotic formula used in the current study on various cell lineages of normal mouse gastric mucosa [28]. This mixture of probiotics was found to mainly upregulate the production of TFF1, TFF2, mucins, and ghrelin and to downregulate pepsinogen expression [28]. Based on these results, the impact of probiotics in preventing and/or treating damage of gastric mucosa is investigated in the current study by using two mouse models oinduced gastric erosions of protocols 1 and 2 (Figure 1). Since ASA was administered by oral gavage, one might argue that the gavage procedure has contributed to the erosive lesions due to stress generated by handling the mice. However, the procedure was done by an expert in handling mice, so the procedure was smooth and quick to avoid discomfort or stress. In addition, the same procedure was carried out in the control mice which received only water by oral gavage. We did not observe erosive lesions in these control mice and their gastric mucosae appeared normal.

Loss of gastric epithelial integrity might result from an imbalance between aggressive and protective factors leading to severe complications ranging from gastritis or mucosal erosions to gastric ulcers. Several studies reported gastric mucosal injury following chronic exposure of the gastric mucosa to ethanol and non-steroidal anti-inflammatory drugs, such as aspirin [38,39]. One of the new strategies to limit gastric mucosal injury is to administer probiotics. However, only few studies reported the therapeutic effect of probiotics on induced gastric ulcer in rats [10,26]. In the present study, we examined the prophylactic and therapeutic effects of probiotics on ethanol/ASA-induced gastric erosions in mice. We have demonstrated that DSF administration pre- or post-induction of erosion significantly reduces the development of gastric erosions. This finding was due to upregulating the secretory activity of mucous cells. Further investigations have shown that DSF administration increases the number of proliferating cells and the production of ghrelin-secreting enteroendocrine cells. Another interesting finding was the restoration of endogenous aggressive factors, such as H^+^,K^+^-ATPase.

A previous meta-analysis study showed that the use of different probiotic bacteria significantly enhances body weight gain in animals and humans [40]. A more recent study also showed that the food intake and body weight of fish significantly increased after probiotic treatment [41]. Body weight gain in general is related to an increase in the appetite which is controlled by ghrelin. In the current study, a significant increase in ghrelin secreting cells was observed in the DSF-pretreated erosion-induced mice. This explains the slight increase, though insignificant, in the bodyweight gain of DSF mice. 

Tissues from mice with induced gastric erosions showed focal areas with superficial or complete disruption of several adjacent gastric glands. Interestingly, DSF-treated or -pretreated mice showed less damage to the gastric mucosa when compared to the gastric erosion group and the few erosive lesions encountered were only superficial. The gastric mucosal thickness was maintained and presented a histological pattern very similar to the control gastric tissues. The present results provide additional evidence supporting the few previous studies which reported that pretreatment with probiotics markedly attenuated induced gastric mucosal injury [25,42,43]. Our studies provide additional explanation towards the stem cell lineages and some cell lineage-specific molecules involved in the action of probiotics.

Using BrdU labeling, we have found that ASA-induced erosion models were associated with increased isthmal cell proliferation which might include the isthmal Mist1-positive stem cells. These cells were also amplified in the isthmus region of gastric glands of the acetic acid-induced ulcer model [44]. We also found that administration of DSF followed or preceded by ASA is associated with an increased number of S-phase labeled cells. Mice used in each experiment of both protocols of this study are not only littermates but also sex- and weight-matched. In control mice, the proliferative cells are few and localized in the narrow isthmus region of the gastric glands, but become more numerous and expanded toward the luminal surface in the erosion-induced and DSF-treated tissues. In the literature, there are conflicting results regarding the effect of probiotics on cell proliferation. In the colon of a normal rat, probiotics administration is not associated with significant change in cell proliferation [45]. However, in an earlier study, counts of mitotic figures in different segments of the small and large intestines of rats following probiotics administration revealed a significant increase in cell proliferation [46]. On the other hand, in rats injected with a carcinogen, probiotics are found to reduce cell proliferation and decrease the incidence of precancerous lesions [47]. Additionally, in vitro, probiotics are found to have anti-proliferative effect against cancer cell lines [48]. In addition, it was reported that a combination therapy including probiotics significantly attenuates tumor growth in a mouse model of colon cancer by promoting late apoptosis and activating pro-apoptotic ERK, not due to inhibition of cell proliferation [49]. Recently, using 3D in vitro and genetically engineered models [50], earlier observations of Lam et al. [22] are confirmed by demonstrating that probiotic *Lactobacilli* stimulate epithelial cell proliferation and provide an answer for a mechanistic pathway via leptin and JAK-STAT signaling pathways. Therefore, results on the effects of probiotics on cell proliferation appear puzzling and still deserve more investigation. This might be due to the fact that these studies explored the proliferative effect of different probiotics formulas on different segments of the gastrointestinal tract using different protocols. An extensive meta-analysis review and further investigations are needed to confirm and explore mechanisms involved in the proliferative effects of probiotics on the gastric epithelium.

The acid-secreting parietal cells complete their differentiation in the vicinity of the isthmal stem/progenitor cells and act as governors for their proliferative and differentiation activities [51]. The current results indicate that while erosion induction upregulates H^+^,K^+^-ATPase in parietal cells, DSF administration before or after the induction of erosions (protocols 1 and 2) contributes to stabilize the amount of H^+^,K^+^-ATPase. This might be linked to the attenuation of the damaging effects of ASA in the DSF-pretreated or –treated mice and also indicates a role for probiotics in maintaining a normal level of acid secretion when the gastric mucosa is exposed to single or multiple insults. This might be due to up-regulation of TFF2 which not only stabilizes the gastric mucous layer [52], but also inhibits acid secretion [53]. 

In the present and previous studies, lectins binding to glycoconjugates have been used as a marker of mucins in surface and neck mucous cells of the gastric glands [31]. This study demonstrates that probiotics administration before or after aspirin insults (protocols 1 and 2) causes a significant increase in the production of mucus. Increased mucus secretion was not surprising and coincides with previously demonstrated increased thickness of mucus layer by probiotic strains: *Streptococcus thermophiles* CRL 1190 [54] and *Bifidobacterium bifidum* BF-1 [55] to respectively alleviate chronic gastritis in mice or acute gastric injury in rats. In a similar study, *Lactobacillus rhamnosus* GG was shown to indirectly stimulate mucus secretion in ethanol-induced gastric mucosal lesions [22]. In vitro studies have also demonstrated an increase in mucin gene expression in large and small intestinal epithelial cell lines [36,56]. In the small intestine, treatment with *Lactobacillus heveticus* R239 has been found to increase the number of mucus-secreting goblet cells [57]. In the colon of a mouse model of dextran-sodium sulfate-induced colitis, the situation was different and it was reported that probiotics had affected neither MUC gene expression nor surface mucous layer formation [58]. Therefore, there are conflicting reports regarding mucus-secreting goblet cells in the small and large intestines. However, in the stomach, it seems that there is an agreement on the stimulating effects of probiotics not only on the expression and secretion of mucus, but also on the production or differentiation of mucous cells.

TFF1 and TFF2 are supportive constituents of the mucous layer on the gastric epithelium [59]. While monomeric TFF1 might protect the gastric mucosa as a scavenger for extracellular reactive oxygen/nitrogen species, the TFF2/MUC6 complex stabilizes the inner layer of the gastric mucous layer [59] and inhibits acid secretion [53]. Therefore, TFFs play critical roles in maintaining mucosal integrity by controlling cell proliferation and migration-associated differentiation [31,53,60]. Consequently, TFFs have been proposed as good therapeutic candidates for several gastrointestinal diseases. Few studies have addressed the impact of probiotic administration on TFFs secretion. Interestingly, our study has demonstrated that the inhibition of gastric mucosal erosions by DSF pretreatment is associated with upregulation of TFF1 and TFF2. It has been reported that upregulation of TFF1 in an inflamed or damaged gastrointestinal tract promotes epithelial restitution to restore the integrity of the epithelial barrier [59]. In addition, the administration of genetically engineered TFF-secreting *Lactococcus lactis* exerted both prophylactic and therapeutic effects in a mouse model of colitis [61].

This study shows that decreased GS-labeled mucus in neck cells is associated with a reduction in pepsinogen immunolabeling in erosion-induced tissues. In DSF-pretreated tissues, there is an increased GS-labeled mucus in neck cells associated with some conservation of the amount of pepsinogen in zymogenic cells. Therefore, the acute insult induced by ASA gavage caused a significant inhibition in the production of both mucin 6 of neck cells and pepsinogen by zymogenic cells which were prevented in mice pretreated with DSF. This might indicate that both neck and zymogenic cells and their intermediate forms (prezymogenic cells) share similar signals involved in their secretory pathway. This is not unexpected since zymogenic cells originate from neck cells which in turn come from the isthmal pre-neck cell progenitors and therefore all three cell types belong to the same lineage [62]. This does not exclude the fact that a small proportion of zymogenic cells originates from their own mitosis [62,63]. In humans, mild to severe inflammatory reaction in the gastric mucosa is also associated with a decrease in pepsinogen level leading to precancerous and cancerous changes [64]. 

Ghrelin represents an important local protective factor in the gastric mucosa mainly due to its anti-inflammatory properties mediated by prostaglandins and inhibition of acid secretion [65,66]. However, it is not well explored whether probiotics pretreatment might modulate ghrelin expression or stem cell production of ghrelin-secreting enteroendocrine cells. Our results have demonstrated that pretreatment with probiotics has slightly, but significantly, increased the number of ghrelin-producing cells in the gastric glands. These results support previous reports in which pretreatment with probiotic bacteria *Escherichia coli* strain Nissle 1917(EcN) attenuated the acute gastric mucosal lesions induced by water immersion and restraint stress through several mechanisms including the secretion of ghrelin [64]. In another study, supplementation of fish larvae with probiotic formula containing *Lactobacillus rhamnosus* significantly increased the ghrelin expression [41]. A recent study has also demonstrated that administration of heat-killed *Lactobacillus brevis* SBC8803 increased ghrelin secretion both in vivo and in vitro [67]. Coherent results were also reported in our previous study and other studies about the effect of probiotics on ghrelin expression in the gastric mucosa [41,67]. Even in children with acute lung injury, the level of serum ghrelin was elevated following the administration of probiotics for 10 days [68]. However, a more recent study has shown that different probiotic strains, including *Bifidobacterium* and *Lactobacillus genera*, attenuate ghrelin-mediated signaling through the ghrelin receptor GHSR-1a [69]. 

## 5. Conclusions and Perspectives

Numerous clinical and experimental studies have explored the beneficial applications of the probiotics for gastrointestinal disorders. In this study, the effects of the multi-strain DSF was investigated in mice with two protocols of ASA-induced gastric erosions. Our findings indicate that DSF exert an ameliorative role against damage of gastric mucosa involving increased stem/progenitor cell proliferation and modulation of several epithelial stem cell progeny: surface mucous, mucous neck, parietal, and enteroendocrine cells. At the molecular level, DSF pretreatment caused an increase in mucins, TFFs, and ghrelin, but a restoration in H^+^,K^+^-ATPase and pepsinogen. Taken together, these data support the beneficial effects of probiotics for gastric mucosal erosions. 

With all these current and previous promising findings, still there are some controversies [70]. On one hand there are not enough studies on the possible side effects of some probiotics and still there are some unanswered questions. Which probiotics are safer or more effective than the others and for which patient or disease? Which age group of people should avoid probiotics? Are probiotics safe for patients with special medical conditions, such as those receiving chemotherapy with weak immune reaction? Which probiotics survive the hostile environment of the stomach? On the other hand, multiple lines of evidence identify probiotics as a beneficial agent for many diseases affecting different body organs and systems. Some probiotic bacteria are known to survive in the acidic environment of the stomach especially when in a multi-strain mixture [7,8,9,71]. The potential use of probiotics is even proposed for COVID-19 patients with gastrointestinal dysbiosis and pneumonia. This might provide an additional evidence for the interesting concept of gut-lung axis [72]. However, studies explaining modes of probiotics action are still few and those explaining specific mechanisms, signaling pathways, and genes involved are needed. Such studies on well-defined diseases in animal models and humans will provide evidence-based clinical use of probiotics in health and disease. 

## Data Availability

The raw data used to generate the figures presented in this study are readily available upon request.
